# Giant Vertical Magnetization Shift Caused by Field-Induced Ferromagnetic Spin Reconfiguration in Ni_50_Mn_36_Ga_14_ Alloy

**DOI:** 10.3390/ma13214701

**Published:** 2020-10-22

**Authors:** Fanghua Tian, Yin Zhang, Chao Zhou, Qizhong Zhao, Zhonghai Yu, Adil Murtaza, Wenliang Zuo, Sen Yang, Xiaoping Song

**Affiliations:** MOE Key Laboratory for Nonequilibrium Synthesis and Modulation of Condensed Matter, School of Physics, Xi’an Jiaotong University, Xi’an 710049, China; tfh2017@xjtu.edu.cn (F.T.); yzhang18@xjtu.edu.cn (Y.Z.); chao.zhou@xjtu.edu.cn (C.Z.); zhaoqizhong@stu.xjtu.edu.cn (Q.Z.); yuzhh1123@stu.xjtu.edu.cn (Z.Y.); adilmurtaza91@mail.xjtu.edu.cn (A.M.); zuowenliang@xjtu.edu.cn (W.Z.); xpsong@xjtu.edu.cn (X.S.)

**Keywords:** Heusler alloy, spin glass, vertical magnetization shift, spin reconfiguration

## Abstract

Vertical magnetization shift (VMS) is a special type of exchange bias effect that may lead to a revolution in future ultrahigh-density magnetic recording technology. However, there are very few reports focusing on the performance of VMS due to the unclear mechanism. In this paper, a giant vertical magnetization shift (M_E_) of 6.34 emu/g is reported in the Ni_50_Mn_36_Ga_14_ alloy. The VMS can be attributed to small ferromagnetic ordered regions formed by spin reconfiguration after field cooling, which are embedded in an antiferromagnetic matrix. The strong cooling-field dependence, temperature dependence, and training effect all corroborate the presence of spin reconfiguration and its role in the VMS. This work can enrich VMS research and increase its potential in practical applications as well.

## 1. Introduction

Since the discovery of the exchange bias effect, it has been utilized to fabricate hard disk drives, spin valves, magnetic tunnel junctions, and other “spin electronic” devices [1,2,3,4]. There are two main types of exchange bias effect research, the horizontal exchange bias effect (EB) and the vertical magnetization shift (VMS). EB is the offset of the hysteresis loop along the field axis, which is observed and studied in many materials [5,6,7,8,9,10,11,12]; VMS is the offset of the hysteresis loop along the magnetization axis, which is only found in a small number of systems [13,14,15,16,17,18]. M_E_ denotes the shift of the center of gravity of the hysteresis loop along the magnetization axis. It is a measure of the average value of magnetizations at the positive and negative measuring fields (M_max+_ and M_max−_, respectively), M_E_ = (M_max+_ + M_max−_)/2 [13,14,15]. For example, Tian et al. obtained an M_E_ = 0.15 emu/g in NiFe_2_O_4_ nanoparticles. The observed VMS is explained in terms of the exchange interaction between the ferrimagnetic phase and the spin glass-like (SG) phase at the interface [13]. Zheng et al. reported an M_E_ = 45 emu/cm^3^ in SrRuO_3_/SrTiO_3_ heterostructures due to the lattice distortion caused by the oxygen deficiency, which modifies the strong hybridization of *p-d* orbitals and perpendicular uniaxial magnetic anisotropy [15]. So far, the reported M_E_ is relatively small, and a comprehensive understanding of the VMS effect is still disputed, restricting the practical applications of VMS.

Ni–Mn–Z (Z = Ga, Sn, In, Sb) Heusler alloys have attracted much attention and have been extensively studied in the past decade [19,20,21], because off-stoichiometric Ni–Mn–Z Heusler alloys contain two mixed phases: (i) the conventional interaction of Mn atoms produces ferromagnetism; (ii) an additional interaction of the Mn atoms produces antiferromagnetism. In some regions, the antiferromagnetic (AFM) interaction becomes stronger than the ferromagnetic (FM) interaction, thereby producing AFM regions, while in other regions, an SG state will occur due to the interaction of the two types of magnetic states at low temperatures [22,23]. Furthermore, over a very large composition range, a reversible martensitic transformation may occur in the alloys, producing complex magnetic properties at low temperatures. The interplay between magnetic and structural degrees of freedom produces many unique functional properties, including a large magnetocaloric effect, giant magnetoresistance, large magnetic-field-induced strains, and large EB [24,25,26,27]. It can be known from the previous literature that in the Ni_50_Mn_25+X_Ga_25−X_ (0 ≤ x ≤ 25) alloy, when x ≤ 15, the alloy enters the spin glass state of the FM and AFM interaction at low temperatures [28,29]. This interaction can lead to the EB effect. For example, Han et al. observed the EB effect both after the field cooling (FC) and zero field cooling (ZFC) process in off-stoichiometric Ni_50_Mn_35_Ga_15_ alloy [29].

In this work, the VMS is studied in the Ni_50_Mn_36_Ga_14_ Heusler alloy. Besides, a systematic investigation of the effects of the temperature and cooling field on M_E_ is analyzed, giving a new way to further understanding and improvement of tunable spintronic devices. This discovery can also enrich the VMS research and increase its potential in practical applications.

## 2. Material and Methods

In this study, bulk polycrystalline Ni_50_Mn_36_Ga_14_ alloy was prepared by arc melting and cast into a copper mold under argon atmosphere. For homogenization, the prepared alloy was sealed in a silica tube furnace and annealed at 1173 K for 24 h. The annealing temperature is generally selected as a temperature slightly below the melting temperature [28]. It is known from the literature that the melting temperature of NiMnGa alloy is about 1300 K [30], so the annealing temperature used in this article was 1173 K. X-ray diffraction (XRD) measurements were performed using a Bruker D8 X-ray diffractometer (Bruker, D8 ADVANCE, Miroslava Minkova, Germany) with Cu Kα1 radiation. The phase transformation temperature was determined by differential scanning calorimetry (DSC, TA Instruments-Waters, Q2000, New Castle, DE, USA) with a heating/cooling rate of 10 K/min. Compositional analysis was performed by means of energy dispersive spectroscopy (EDS, JEOL JSM-7000F, Tokyo, Japan) of the samples. The magnetic properties including, the magnetic hysteresis (M-H) loops, magnetization–temperature (M–T) curves, and AC susceptibility were measured using a superconducting quantum interference device (SQUID) magnetometer (Quantum Design, MPMS-XL-5, San Diego, CA, USA).

## 3. Results

**Figure 1:**
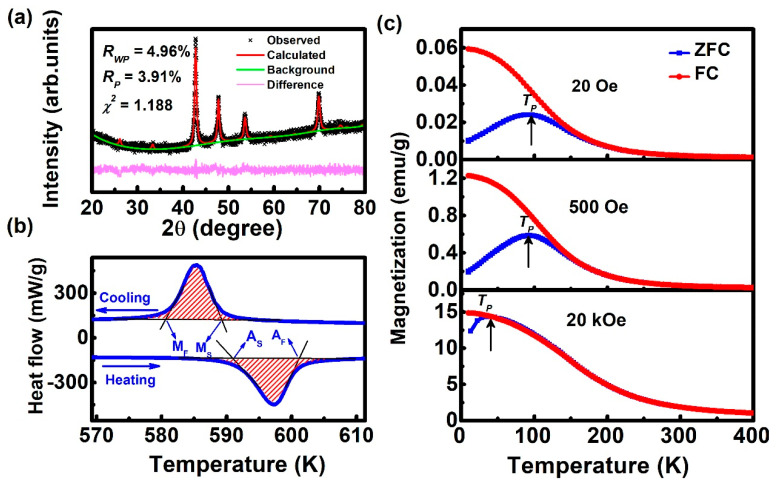
(**a**) XRD pattern of the Ni_50_Mn_36_Ga_14_ alloy at room temperature. (**b**) Martensitic transformation behavior of the alloy measured by DSC using a cooling/heating rate of 10 K/min. (**c**) Temperature dependence of the magnetization curves for an applied magnetic field of 20 Oe, 500 Oe, and 20 kOe for zero field cooling (ZFC) and FC, respectively.

The XRD pattern of the bulk Ni_50_Mn_36_Ga_14_ alloy at room temperature is shown in Figure 1a. The observed pattern is consistent with a single tetragonal L1_0_ structure (martensitic phase) [31,32]. The calculated results are based on the tetragonal structure with space group P4/mmm, and the small values of the reliability factors (*R_P_*: profile factor, *R_WP_*: weighted profile factor, *χ^2^*: goodness of fit) of the Rietveld refinements suggest the good agreement between the experimental and calculated diffraction patterns of the sample. The alloy has the following lattice parameters at room temperature: a = b = 7.6077 Å and c = 6.7976 Å. The temperature hysteresis of heat flow transition peaks obtained through the DSC measurements is shown in Figure 1b. The start and the end of the transformation temperatures have been determined by the intersection of a baseline and the tangents to each peak. Its characteristic martensitic phase transition temperatures, i.e., the austenite start and finish temperatures (*A_s_* and *A_f_*) and the martensite start and finish temperatures (*M_s_* and *M_f_*), were determined to be 591 ± 0.2, 601 ± 0.2, 589 ± 0.2, and 580 ± 0.2 K, respectively. The sample undergoes the endothermic process with a phase transformation enthalpy of 1.85 J/g upon heating, and upon cooling, it undergoes the exothermic process with an enthalpy of 1.83 J/g. The enthalpy difference between them is only about 0.02 J/g, showing good reversibility of the phase transformation. The first-order nature of the phase transition was also confirmed from the observed temperature hysteresis (12 K) of heat flow transition peaks. In addition, Figure S1 plots the DSC measurement results of the Ni_50_Mn_36_Ga_14_ alloy for 15 consecutive heating and cooling thermal cycles. It can be clearly seen that after 15 cycles, the martensitic transformation peak has only moved about 1.5 K compared to the first cycle, which indicates that the phase transition of the Ni_50_Mn_36_Ga_14_ alloy has good reproducibility. From the above XRD and DSC results, it can be concluded that the alloy is a martensitic phase with L1_0_ structure at room temperature.

Figure 1c presents the temperature dependence of magnetization measured under ZFC and FC of the Ni_50_Mn_36_Ga_14_ alloy under different applied magnetic fields, and the rate is 3 K/min. In the case of the ZFC curve, the magnetization first increases and then decreases with decreasing temperature and reaches a maximum at ~110 K. On the other hand, the observed FC curve shows an FM-like behavior. The large splitting between the ZFC and FC curves at low temperatures indicates an inhomogeneous magnetic state (i.e., an SG or AFM state with FM regions) in this regime [5,8,33]. The temperature dependence of magnetization was also measured under an applied magnetic field of 500 Oe and 20 kOe (shown in the middle and bottom panel of Figure 1c, respectively). In this case, the bifurcation of the FC and ZFC curves appears after the temperature decreased to 100 K and 35 K, respectively, indicating that after a high field cooling, SG behaves like FM. Such a feature is also typical for glassy magnetic states in the case of a martensitic phase transformation [3].

At the same time, the element ratio of the annealed alloy was analyzed by EDS. The result is given in Figure S2, which illustrates analytical spectra and the corresponding alloy composition. It is seen that the atomic percentage (at %) of Ni, Mn, and Ga is 51.2 ± 1, 34.5 ± 1, and 14.3 ± 1, respectively. It deviates from the nominal composition (Ni_50_Mn_36_Ga_14_) at an acceptable level.

For comparison, the XRD, DSC, and MT of the as-cast were also studied, as shown in Figure S2. Figure S2a shows DSC scans for as-cast and annealed Ni_50_Mn_36_Ga_14_. In both the as-cast and annealed alloys, a couple of peaks indicate a martensitic transformation during the cooling and heating, but the peak is much broader in the as-cast alloy. Thus, annealing helps to achieve compositional homogeneity. This is because, during annealing, the elements in the alloy can diffuse more sufficiently to reduce the inhomogeneity of the chemical composition (segregation). X-ray diffraction patterns of both alloys recorded at room temperature are shown in Figure S2b. The structure of both alloys is almost identical, except for slight differences in the peak intensities and angles. The magnetic moment measurements revealed that upon annealing, a significant increase of magnetization occurs (Figure S2c). In the following, the annealed samples are used for all measurements.

**Figure 2:**
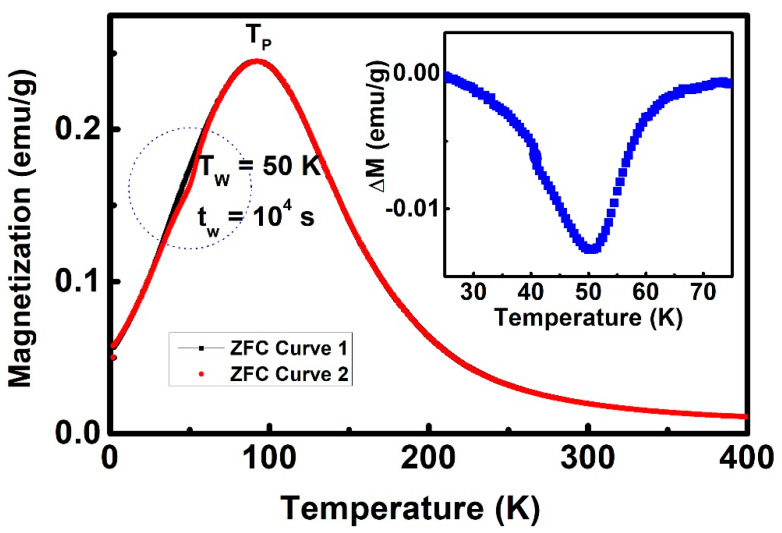
Temperature dependence of ZFC magnetization with (red line) and without (black line) the stop-and-wait protocol (at T = 50 K, t_W_ = 10^4^ s) measured under an external 200 Oe field. The inset shows the difference between Curve 1 and Curve 2 around T = 50 K.

An obvious feature of SG is that it has a memory effect, which can be tested through the stop-and-wait protocol [34]. In the SG state, when the temperature is lower than T_P_, the size of the glass will grow with the waiting time even under the ZFC process, showing a good memory effect. Figure 2 shows the stop-and-wait protocol test of the Ni_50_Mn_36_Ga_14_ alloy. First, cool the sample from 400 K to 2 K without an external field, and then, record the change of the magnetization curve with temperature (Curve 1, as the reference curve) while under a 200 Oe external field during heating. Then, add the stop-and-wait procedure: first cool the sample from 400 K to the intermediate stop temperature T_W_ = 50 K (T_W_ < T_P_) under the ZFC process, where the sample waits for t = 10^4^ s, and then, further cool to 2 K; the data were recorded during the heating process with an external 200 Oe magnetic field as Curve 1 (Curve 2). As shown in Figure 2, the magnetization of the two magnetization curves basically overlaps in all other temperature regions, except for the inconsistency near 50 K. The difference between Curves 2 and 1 is shown in the inset of Figure 2. It can be clearly observed that the alloy memory effect is around 50 K. This memory effect confirms that martensite exhibits the SG behavior at a temperature lower than T_P_. Moreover, the temperature dependence of the electrical resistivity is presented in Figure S4. The resistivity shows an insulator behavior at higher temperatures, but around T_P_, an insulator-metal transition occurs, which may be ascribed to the effect of spin-disorder scattering of frozen spins in the magnetic inhomogeneous SG state [35].

**Figure 3:**
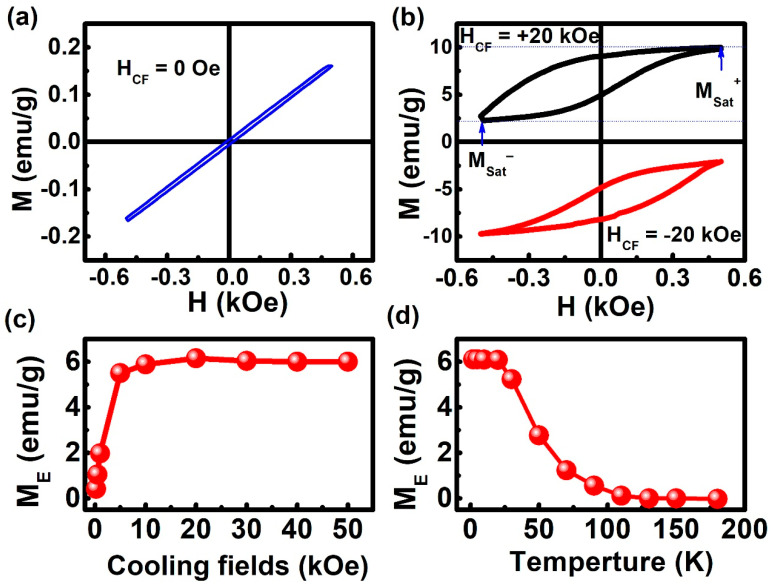
(**a**,**b**) Magnetic hysteresis (M-H) loops obtained for the Ni_50_Mn_36_Ga_14_ alloy at different cooling fields (0 Oe, ±20 kOe). (**c**) M_E_ as a function of _Hmax_ in Ni_50_Mn_36_Ga_14_ at 2 K after different H_CF_. (**d**) M_E_ as a function of temperature in Ni_50_Mn_36_Ga_14_ after 20 kOe.

Figure 3a,b show the magnetic hysteresis (M-H) loops measured for the prepared Ni_50_Mn_36_Ga_14_ alloy after the ZFC and FC. The M-H loops were recorded at different temperatures and magnetic fields applied in the range from −0.5 kOe to +0.5 kOe after the ZFC and FC from 400 K. As shown in Figure 3a, VMS is not observed at ZFC. This is because at ZFC, the FM clusters are randomly orientated. For the Ni_50_Mn_36_Ga_14_ alloy, the M-H loops show an M_E_ = 6.34 emu/g along with the same direction as the cooling field (shown in Figure 3b), i.e., positive cooling field (H_CF_) results in an upward M_E_. This is a typical signature of the system over energy barriers and stabilizes a spin-reconfigured state so that a magnetization-shifted hysteresis loop occurs in the field-cooled state [5]. Further, it is symmetrically opposite with respect to the M-H performed under H_CF_ = −20 kOe, indicating that the observed VMS did not originate from the non-saturated minor loop.

As shown in Figure 3c, the calculated variation of M_E_ initially increased monotonically with increasing cooling field and stabilized after 20 kOe. The M_E_ value increased with an increasing cooling field. This is because the degree of alignment of FM clusters enhanced with the cooling field, leading to a unidirectional exchange anisotropy. The spins at the interface exert a microscopic torque, and AFM clusters may restrict themselves to the FM clusters. When the cooling field reached 20 kOe, the unidirectional FM anisotropy reached its maximum, and the maximum VMS was observed (Figure 3c).

The dependence of M_E_ on the temperature and cooling field was also investigated in this study. Figure 3d shows the temperature dependence of M_E_ for the Ni_50_Mn_36_Ga_14_ alloy under H_CF_ = 20 kOe from 400 K. M_E_ decreased with increasing temperature and reached zero at 100 K. This can be explained by the reduction in unidirectional FM anisotropy with increasing temperature.

**Figure 4:**
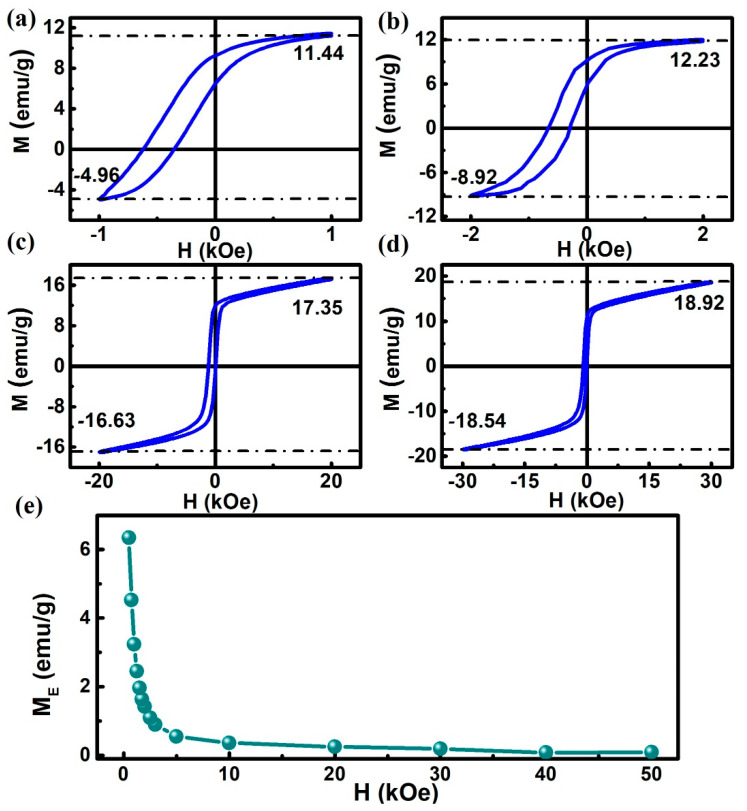
(**a**–**d**) M-H loops obtained for the Ni_50_Mn_36_Ga_14_ alloy at different measuring fields at H_CF_ = 20 kOe. (**e**) M_E_ as a function of measuring fields in Ni_50_Mn_36_Ga_14_ under a cooling field of 20 kOe.

The magnitude of the measurement field has a significant influence on the interaction between FM and AFM. Figure 4 shows the hysteresis loop of the sample at different measuring fields and the trend of M_E_ changing with the measuring field at 2 K under = 20 kOe. As shown in Figure 4a–d, even if the measuring field is increased to 30 kOe, the VMS phenomenon still exists, although the VMS behavior becomes weaker at the large measuring field. Figure 4e plots the variation of M_E_ with the magnitude of the measuring field, and it can be illustrated that the M_E_ decreases monotonously with the increase of the measurement field. At the same time, it is worth noting that the VMS behavior is always accompanied by a conventional EB effect, which has been reported in other systems [13,14,15,16,17,18,36]. This is because the physical origin of the two phenomena in the same system is the same [36].

**Figure 5:**
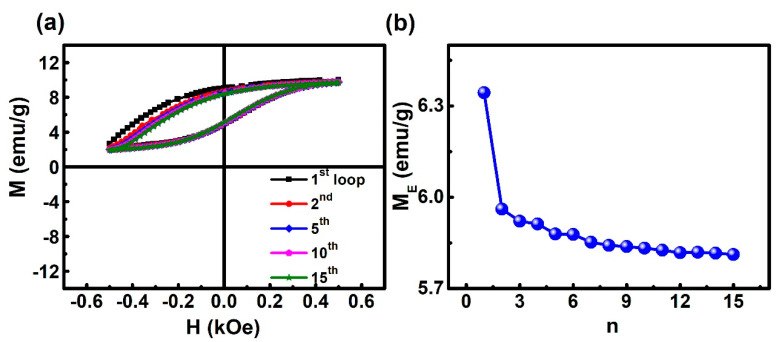
(**a**) Training effect of the Ni_50_Mn_36_Ga_14_ alloy. hysteresis loops measured at temperature T = 2 K after field cooling (FC) under 20 kOe from 400 down to 2 K. (**b**) M_E_ as a function of field cycle number *n*.

The training effect is an inherent characteristic of the exchange bias effect (including EB and VMS), and the magnetic state of the material can be studied through the training effect. Figure 5 shows the hysteresis loops measured from the first to 15th of the sample after FC down to 2 K under 20 kOe. As shown in Figure 5, after the first few cycles, the value of M_E_ gradually decreases with the number of cycles (n). The first loop exhibits an M_E_ of ~6.34 emu/g. This value decreases to 5.82 emu/g when *n* = 11. At n > 11, M_E_ almost keeps constant. Such a tendency of M_E_ to change with *n* is similar to other literature [37,38]. M_E_ first decreases with *n* and then becomes stable with a further increase of n being mainly due to the reconfiguration of some spins during the initial cycles and their stabilization in the subsequent cycles.

**Figure 6:**
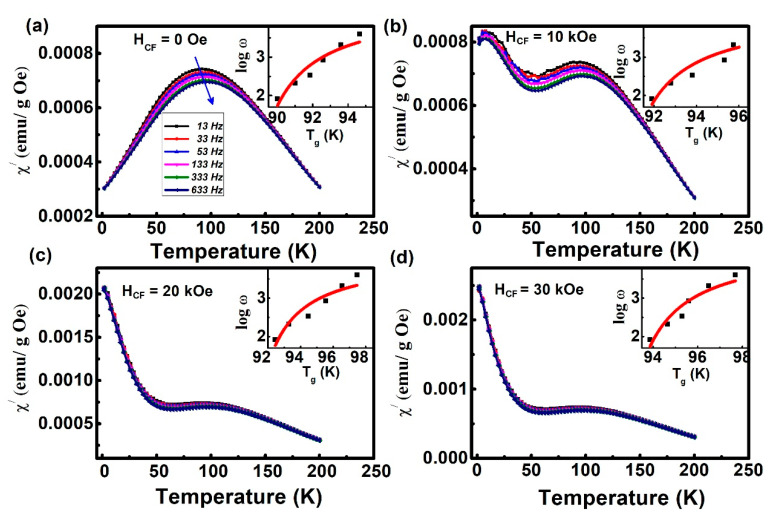
The temperature dependence of the real part of AC susceptibility is measured at different frequencies with an AC magnetic field of 2 Oe under different cooling field 0 Oe (**a**), 10 kOe (**b**), 20 kOe (**c**), 30 kOe (**d**). The inset shows the correlation between angular frequency and temperature ($T_{f}$). The frequency (ω) dispersion behavior of temperature ($T_{g}$) conforms to the Vogel–Fulcher (V–F) relationship. The arrows indicate the direction of increasing frequencies.

To further investigate the origin of the spin reconfiguration in the glassy magnetic state, the temperature dependence of the real part (χ’) of AC susceptibility was recorded at different frequencies (13, 33, 53, 133, 333, and 633 Hz) after different cooling fields, and the resulting curves are shown in Figure 6. It is illustrated in Figure 5 that at all cooling fields, the χ’ peak shifts towards higher temperatures, and its value decreases as the frequency increases.

The Vogel–Fulcher (V-F) relationship could be employed to analyze the SG-like behavior [39,40] of the sample,

(1) $${\omega \;=\;\omega }_{0\;}{\exp[-E}_{a\;}{/\;k}_{B}{\;(T}_{f}{-T}_{0})]$$

where ω  is the measured angular frequency; $\omega_{0\;}$ is the characteristic frequency of SG; Ea is the activation energy of SG; k_B_ is the Boltzmann constant; T_f_ is the peak temperature on the χ’-T curves; T_0_ is the V–F temperature that describes the interaction among SG clusters. $\tau_{0}\;=\;2\pi \;/\;\omega_{0}$ is the microscopic relaxation time related to the nanoclusters in SG. From the previous literature, τ_0_ varies from 10^−5^–10^−12^ for SG [24,41]. Through fitting, Ea, T_0_, and $\tau_{0}$ were all obtained, as given in Table 1. It is obvious from Table 1 that all the characteristic parameters fall in the typical value ranges of SG. As the cooling field increases, τ_0_ becomes larger. Note that the size of the nanoclusters in SG is smaller, and τ_0_ would be smaller. Thus, the size of the FM cluster in SG increases as the cooling field increases, which suggests spin reconfiguration with the cooling field increasing indeed occurs [5]. Combining the results given in Figure 3, it can be speculated that the spin reconfiguration of FM clusters with the cooling field plays an essential role in the VMS behavior of the sample.

## 4. Discussion

**Figure 7:**
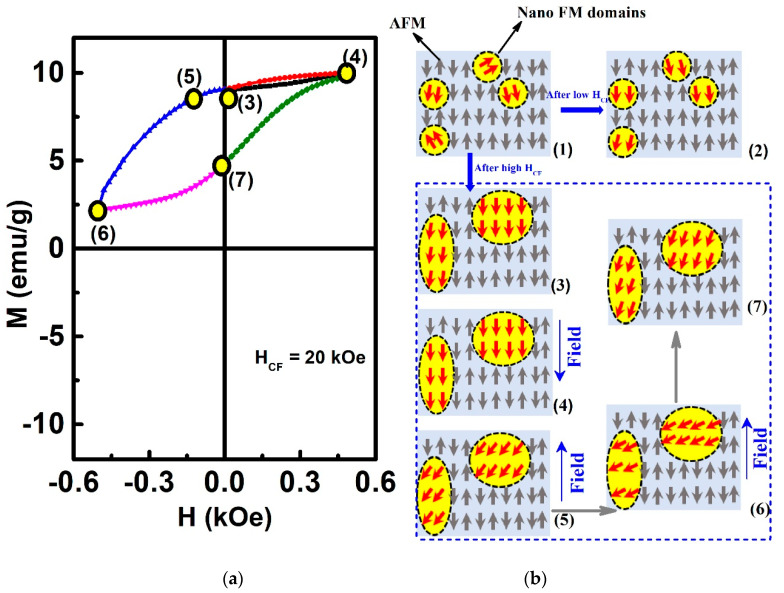
(**a**) Hysteresis loops after H_CF_ = 20 kOe at 2 K. (**b**) Schematic illustration of the effect of the cooling field on the relative proportions of the FM and AFM, where FM and AFM represent the ferromagnetic domain and antiferromagnetic matrix, respectively.

Figure 7 thus schematically illustrates the proposed evolution process of the magnetic structure of the sample giving rise to the VMS phenomenon. As the sample is cooled from 400 K to 2 K under a cooling field, the initially randomly oriented FM spins (Figure 7(b1)) are reconfigured to the direction of the cooling field, and the size of FM domains increases with the increase of the cooling field. Removing the cooling field, most of the FM domains would keep the field direction, and some of them deviate from the field direction by a small angle (Figure 7b (2) and (3)) [5]

Starting from the FM spin state given by high field cooling (Figure 7b (3)), when applying a magnetic field (the same as the cooling field direction), all the FM spins would orient along the field direction (Figure 7b (4)). As the field reduces to zero, some of the FM spins would deviate from a positive direction. When further applying the opposite magnetic field, the FM spins would try to orient towards the negative direction. Still, due to the interaction among the AFM matrix and the FM spins, the FM spins would deviate from the positive direction only by a small angle (Figure 7b (5)) and Figure 7b (6)). Thus, the average magnetization of the sample still keeps a positive value. When reducing the negative field to zero, the FM spins would deviate from the positive direction by a smaller angle, and thus, the average magnetization of the sample becomes a larger positive value as compared to that at the maximum opposite field (Figure 7b (7)). In other words, the materials behave as if an extra (internal) biasing field exists, which leads to the hysteresis loop shifting in the magnetization axis [13,14,15,16].

Note that a minor loop might induce a similar magnetization shift [42]. However, the M-H loops measured at high magnetic fields such as 30 kOe still show a similar VMS behavior, although the M_E_ value is smaller. Therefore, the possibility that the above VMS phenomenon is caused by the small external magnetic field (500 Oe) used here could be excluded.

## 5. Conclusions

In summary, for the bulk Ni_50_Mn_36_Ga_14_ alloy, a giant M_E_ of 6.34 emu/g was observed when the sample was cooled down from 400 K under H_CF_ = 20 kOe. Moreover, M_E_ depended on the magnitude of the cooling field. This is mainly due to the difference in the FM cluster size formed by the reconfiguration of FM spins. At the same time, *M_E_* decreased with increasing temperature, which could be attributed to the change in FM anisotropy with increasing temperature. This result opens a new direction to achieve the VMS, indicating a way to fabricate VMS samples.

## Figures and Tables

**Table 1:** Values of the parameters obtained by fitting the experimental data to Equation (1).

Cooling Field (kOe)	T_0_ (K)	τ_0_ (s)	E_a_/K_B_ (K)
0	87.1	5.1 × 10^−9^	621.5
10	89.3	9.7 × 10^−7^	648.2
20	90.1	8.4 × 10^−6^	669.1
30	91.7	3.5 × 10^−5^	752.8

## Supplementary Materials

The following are available online at https://www.mdpi.com/1996-1944/13/21/4701/s1: Figure S1: Fifteen cyclic DSC responses of the Ni50Mn36Ga14 alloy, Figure S2: (a) The scanning electron microscopy image of the Ni50Mn36Ga14 alloy. (b) EDS analysis of the element ratio of the annealed alloy, Figure S3: (a) Martensitic transformation behavior of the alloy as-cast and annealed measured by DSC using a cooling/heating rate of 10 K/min. (b) XRD pattern of alloys at room temperature. (c) Temperature dependence of the magnetization curves for alloys in the as-cast and annealed at the applied magnetic field of 500 Oe for ZFC and FC, respectively, Figure S4: Temperature dependence of resistivity at zero field of the alloy.
