# Supplementary material for: Giant Vertical Magnetization Shift Caused by Field-Induced Ferromagnetic Spin Reconfiguration in Ni50Mn36Ga14 Alloy
Source: Materials (Basel). 2020 Oct 22;13(21):4701. doi: 10.3390/ma13214701 (PMC7659958; doi:10.3390/ma13214701)
Supplement: Supplementary file 1 [file materials-13-04701-s001.pdf]

materials

MDPI

Supplementary Materials

Giant vertical magnetization shift caused by field-induced ferromagnetic spin reconfiguration in $Ni_{50}Mn_{36}Ga_{14}$ alloy

Fanghua Tian, Yin Zhang, Chao Zhou, Qizhong Zhao, Zhonghai Yu, Adil Murtaza, Wenliang Zuo, Sen Yang, * and Xiaoping Song

MOE Key Laboratory for Nonequilibrium Synthesis and Modulation of Condensed Matter, School of Physics, Xi'an Jiaotong University, Xi'an 710049, China; tfh2017@xjtu.edu.cn (F.T.); yzhang18@xjtu.edu.cn (Y.Z.); chao.zhou@xjtu.edu.cn (C.Z.); zhaoqizhong@stu.xjtu.edu.cn (Q.Z.); yuzhh1123@stu.xjtu.edu.cn (Z.Y.); adilmurtaza91@mail.xjtu.edu.cn (A.M.); zuowenliang@xjtu.edu.cn (W.Z.); xpsong@xjtu.edu.cn (X.S.)

*Correspondence: yang.sen@xjtu.edu.cn

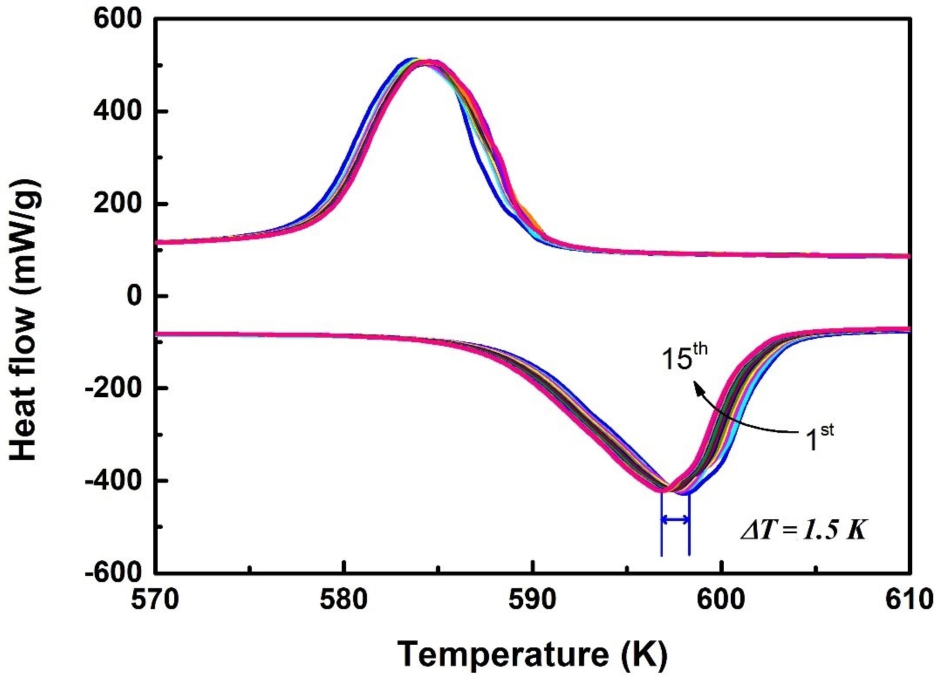

Figure S1. Fifteen cyclic DSC responses of $Ni_{50}Mn_{36}Ga_{14}$ alloy.

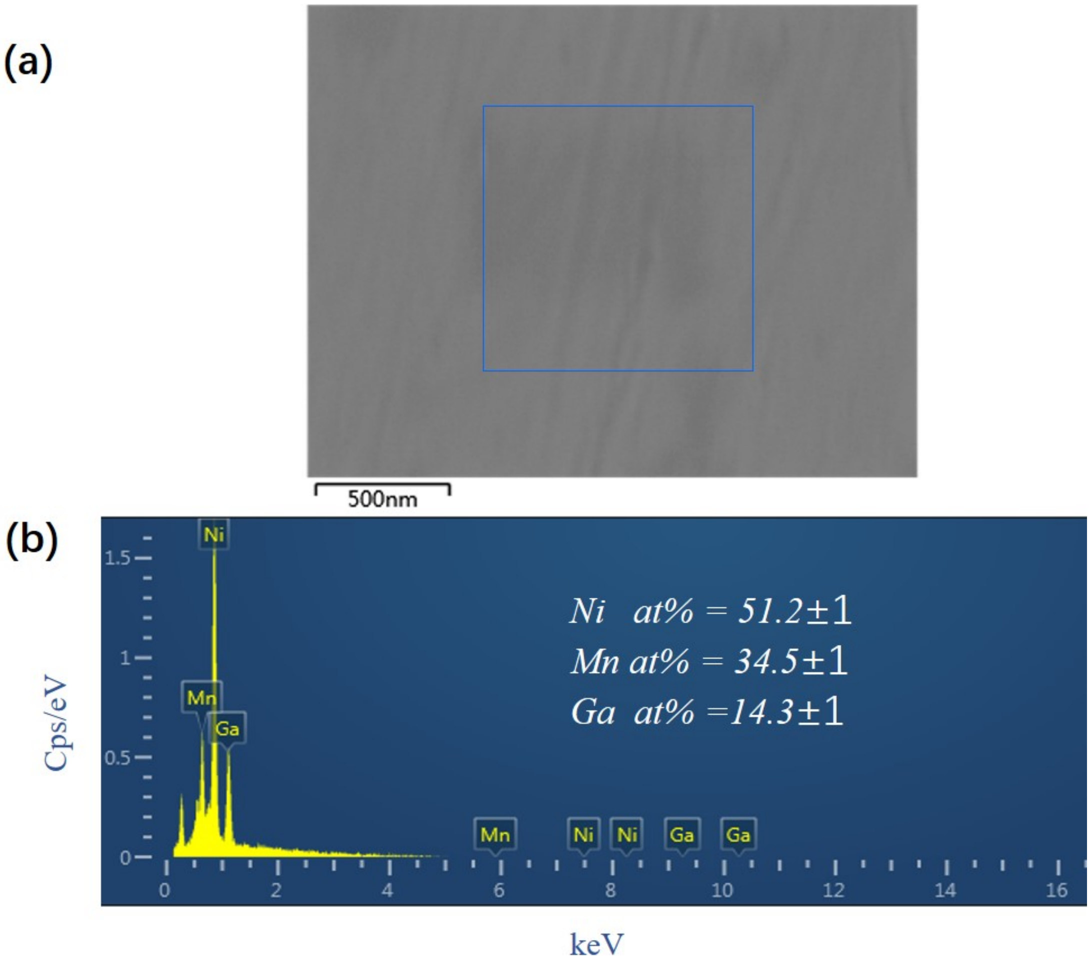

Figure S2. (**a**) The scanning electron microscopy image for $Ni_{50}Mn_{36}Ga_{14}$ alloy. (**b**) EDS analyzed the element ratio of the annealed alloy.

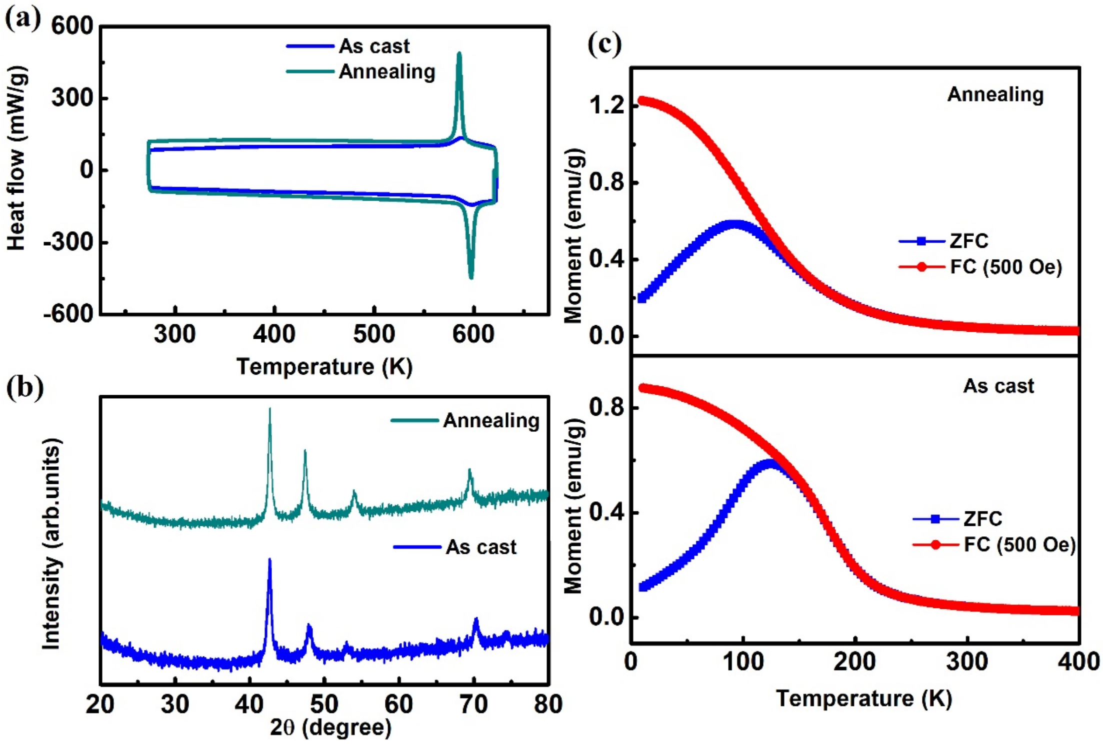

Figure S3. (**a**) Martensitic transformation behavior of alloy in the as-cast and annealed measured by DSC using a cooling/heating rate of 10 K/min. (**b**) XRD pattern of alloys at room temperature. (**c**) Temperature dependence of magnetization curves for alloys in the as-cast and annealed at the applied magnetic field of 500 Oe for ZFC and FC, respectively.

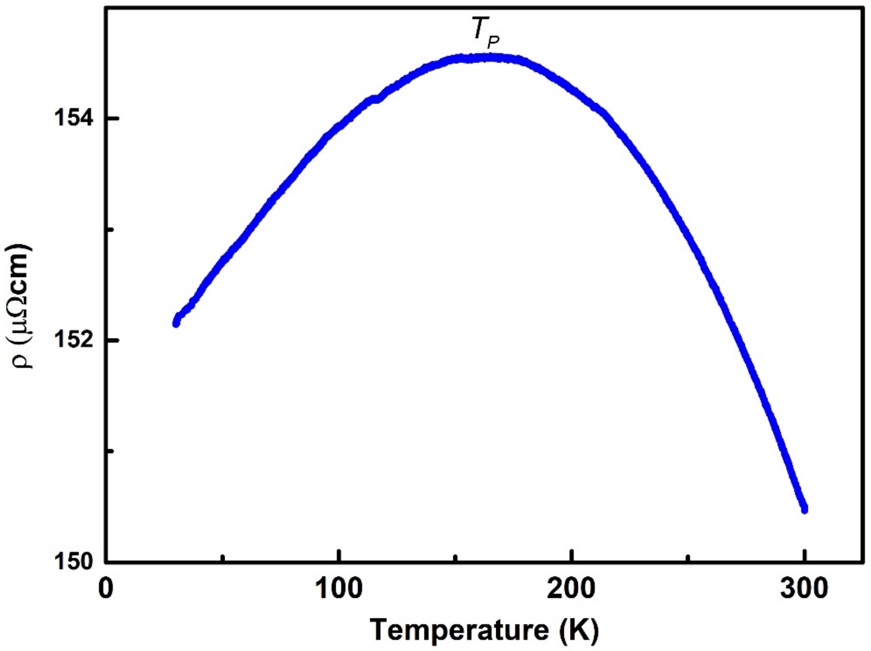

Figure S4. Temperature dependence of resistivity at zero field of alloy.

© 2020 by the authors. Submitted for possible open access publication under the terms and conditions of the Creative Commons Attribution (CC BY) license (http://creativecommons.org/licenses/by/4.0/).
